# Supplementary material for: Learning curve and surgical outcome of robotic assisted colorectal surgery with ERAS program
Source: Sci Rep. 2022 Nov 29;12:20566. doi: 10.1038/s41598-022-24665-w (PMC9709162; doi:10.1038/s41598-022-24665-w)
Supplement: Supplementary file 2 — Supplementary Information 2. [file 41598_2022_24665_MOESM2_ESM.zip › RAL Raw data-Ñ[▒K/058/OP N_ñw╝╨░O▒Kñσ.pdf]

W52 / W52

臺中榮民總醫院
手術室護理記錄

[redacted]

基本資料

- 室別： 開刀房-17室，手術科別： CRS，手術類別： 常規，日期： 2021/01/28
- 進入時間： 0730 ； 麻醉開始時間： 0810 ； Time Out時間： 0955 ； 劃刀時間： 0956 ； 手術結束時間： 1510 送出時間： 1555 ；
- 麻醉方式： GE
  診斷：Malignant tumor of rectum
  手術名稱：Robot Assisted Low Anterior Resection
  手術局部用藥：nil
  術前預防性抗生素：
  - 抗生素名稱一： Veterin 1GM IVD由麻醉人員依醫囑給予
  - 時間： 0950
  - 上次簽章: 廖子晴

  ，追加預防性抗生素：
- 手術醫師： 蔣鋒帆，林陳彥
- 麻醉醫師： VS 齊光彪
-
  - 刷手護士A ： 張家瑋，時間： 0730，刷手護士B ： 羅媗鈴，時間： 0730，接替刷手一： 陳宥廷，時間： 1500
  - 流動護士A ： 廖子晴，時間： 0730，接替流動一： 邱文琇，時間： 1500

皮膚狀況

A-V Shunt： 無
術前： 完整
　記錄者： 上次簽章: 廖子晴
術後： 完整
　記錄者： 上次簽章: 邱文琇

手術準備

手術臥式 單一手術臥式

-
  - 手術臥式： 截石臥式
  - 使用附件： 溫毯 38 ℃，頭圈，腳架
  - 皮膚準備： 10%Aq Betadine，Hibiscrub，2% Chlorhexidine
  - 電燒： 使用電燒: 電極片位置： 右大腿，記錄者： 上次簽章: 廖子晴

止血帶T： 無

植入物

- 植入物名稱/號碼/數量/部位：GIA 80-4.8mm *1 /rectum
  EndoWrist Stapler 45-4.3mm *3 /rectum

，記錄者： 上次簽章: 廖子晴

標本送檢

- 細菌培養 無
- 細胞學 無
- 冰凍切片 無
- 病理標本
  組織名稱一：rectum
  ，
  組織名稱二：IMA root
  ，
  組織名稱三：distal cut end
- 截肢 無

標本交付 標本： 2 袋，標本： 0 盒，標本： 1 瓶，細胞學： 0 管，Culture Tube： 0 管，被交付醫師： CR 林陳彥，

- 記錄者： 上次簽章: 邱文琇
- 時間： 1510

傷口術後數料

傷口數料： 傷口數料： 無

- 請於圖上標示部位：

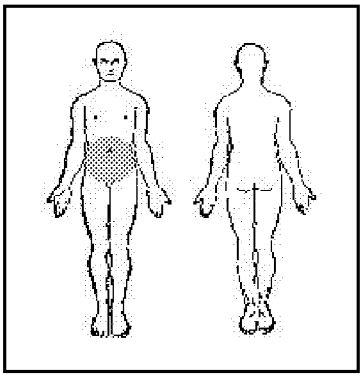

導管

導管	天數	導管資訊	插入日期	插入時間	更換日期	拔除日期	拔除時間	拔除原因	預計拔除/更換	預計拔除/更換日期
Epidural PCA(1)	2	部位:脊髓;	2021/01/28	0820						
Rod,Stoma Supporting (1)	2	部位:迴腸;	2021/01/28	1400						
Double Lumen CVC(1)	2	號碼:8 fr; 深度:14cm; 部位:jugular (右); 象限:RU;	2021/01/28	0840						

用物計數

- 手術中出血量： minimal c.c.
- 手術中尿量： 850 c.c.
-
  - 其它： 無

用物計數： 多切口同時縫合

- 1.器械： 正確，2.縫針： 正確，3.刀片： 正確，4.紗布： 正確，5.腹部墊： 未使用，6.紗球： 未使用，7.PEANUTS ： 未使用，8.紗條： 未使用，9.絲帶： 未使用，10.針頭： 未使用，11.Vessel loop： 未使用，12.Boots： 未使用，13.Jelco： 未使用，14.棉條： 未使用，
  - 第一次：
    - 刷手護士 ： 周筱涵
    - 流動護士： 廖子晴
    - 時間： 1420

  ,
  - 第二次：
    - 刷手護士 ： 周筱涵
    - 流動護士： 廖子晴
    - 時間： 1450

  ,
  - 第三次：
    - 刷手護士 ： 周筱涵
    - 流動護士： 廖子晴
    - 時間： 1455

備註

備註：腹腔鏡no.5

- 記錄者： 上次簽章: 廖子晴

- 時間： 0955

填寫護理人員_廖子晴

正式摘要

	W52 / W52

doctorRank:B
